# Characterization of genes in the *ASYMMETRIC LEAVES2/LATERAL ORGAN BOUNDARIES* (*AS2/LOB*) family in *Arabidopsis thaliana*, and functional and molecular comparisons between *AS2* and other family members

**DOI:** 10.1111/j.1365-313X.2009.03797.x

**Published:** 2009-03-02

**Authors:** Yoko Matsumura, Hidekazu Iwakawa, Yasunori Machida, Chiyoko Machida

**Affiliations:** 1 Plant Biology Research Center, Chubu University1200 Matsumoto-cho, Kasugai, Aichi 487-8501, Japan; 2 Division of Biological Science, Graduate School of Science, Nagoya UniversityFuro-cho, Chikusa-ku, Nagoya 464-8602, Japan; 3 College of Bioscience and Biotechnology, Chubu University1200 Matsumoto-cho, Kasugai, Aichi 487-8501, Japan

**Keywords:** *ASYMMETRIC LEAVES2* (*AS2*), *AS2-like/LOB domain* (*ASL/LBD*) gene, *AS2/LOB* family, AS2/LOB domain, plant development

## Abstract

The *ASYMMETRIC LEAVES2* (*AS2*) gene is required for the generation of the flat and symmetrical shape of the leaf lamina in Arabidopsis. *AS2* encodes a plant-specific protein with an AS2/LATERAL ORGAN BOUNDARIES (AS2/LOB) domain that includes a cysteine repeat, a conserved single glycine residue and a leucine-zipper-like sequence in its amino-terminal half. The Arabidopsis genome contains 42 genes, including *AS2*, that encode proteins with an AS2/LOB domain in their amino-terminal halves, and these genes constitute the *AS2/LOB* gene family. In the present study, we cloned and characterized cDNAs that covered the putative coding regions of all members of this family, and investigated patterns of transcription systematically in Arabidopsis plants. Comparisons among amino acid sequences that had been deduced from the cloned cDNAs revealed eight groups of genes, with two or three members each, and high degrees of identity among entire amino acid sequences, suggesting that some members of the *AS2/LOB* family might have redundant function(s). Moreover, no member of the family exhibited significant similarity, in terms of the deduced amino acid sequence of the carboxy-terminal half, to AS2. Results of domain swapping between *AS2* and other members of the family showed that the AS2/LOB domain of AS2 cannot be functionally replaced by those of other members of the family, and that only a few dissimilarities among respective amino acid residues of the AS2/LOB domain of AS2 and those of other members are important for the specific functions of AS2.

## Introduction

Loss-of-function mutations in the *ASYMMETRIC LEAVES2* (*AS2*) gene in Arabidopsis result in various defects in leaf development, such as the formation of asymmetrically lobed and downwardly curled leaves, the formation of leaflet-like structures from petioles, the generation of an abnormal venation system, and the ectopic expression of class 1 *KNOX* genes in the leaves (Rédei and Hirono, 1964; Ori *et al.*, 2000; Semiarti *et al.*, 2001; Byrne *et al.*, 2002; Xu *et al.*, 2003). Moreover, it has been suggested that *AS2* might be involved in the determination of adaxial-abaxial polarity in leaf primodia (Byrne *et al.*, 2002; Xu *et al.*, 2003; Lin *et al.*, 2003; Ueno *et al.*, 2007; Iwakawa *et al.*, 2007). Loss-of-function mutations in *ASYMMETRIC LEAVES1* (*AS1*), which encodes an MYB domain (recently defined as the SANT domain; Byrne *et al.*, 2003), result in leaf-lamina phenotypes that are similar to those of *as2* plants (Semiarti *et al.*, 2001), and specific phenotypic features generated by the overexpression of *AS2* are reversed by mutations in *AS1*, suggesting that AS1 and AS2 function within, at least, a single pathway or overlapping pathways (Lin *et al.*, 2003; Xu *et al.*, 2003; Iwakawa *et al.*, 2007). The AS2 protein is concentrated in a subnuclear body that is adjacent to nucleoli, and is designated as the AS2 body (Ueno *et al.*, 2007). AS1 is also concentrated in this body via a process that depends on the presence of AS2.

The *AS2* gene encodes a plant-specific protein with a domain that consists of a cysteine repeat (the C-motif), a conserved glycine residue and a leucine-zipper-like sequence in its amino-terminal (N-terminal) half (Iwakawa *et al.*, 2002; Machida *et al.*, 2003). This domain has been designated the AS2 domain or the LATERAL ORGAN BOUNDARIES (LOB) domain (Iwakawa *et al.*, 2002; Shuai *et al.*, 2002; hereafter designated as the AS2/LOB domain). A base substitution mutation in the conserved glycine codon of *AS2* (*as2-5*) results in the typical phenotypic changes observed in various *as2* mutants (Semiarti *et al.*, 2001; Iwakawa *et al.*, 2002). Thus, this residue appears to be essential for the functions of AS2. As another mutant allele, *as2-4*, in which there is a mutation in the region encoding the carboxy-terminal (C-terminal) half of AS2, also causes the typical mutant phenotype, it is clear that the C-terminal half also plays a role in the functioning of AS2.

The Arabidopsis genome contains 42 genes, including *AS2*, that encode putative proteins with an AS2/LOB domain in their N-terminal halves, and the amino acid sequences of the N-terminal halves are significantly conserved among these genes, which together form the so-called *AS2/LOB* gene family. The four cysteine residues in the C-motif and the conserved glycine residue are perfectly conserved in the putative products of all of the genes in this family. By contrast, amino acid residues in the leucine-zipper-like sequences have diverged considerably. Genes in the *AS2/LOB* family are designated as *AS2-like* (*ASL*) genes or *LOB domain* (*LBD*) genes (hereafter referred to as *ASL/LBD* genes), and can be divided into at least two classes, class I and class II, on the basis of the deduced amino acid sequences of their AS2/LOB domains (Iwakawa *et al.*, 2002; Shuai *et al.*, 2002). It has been reported that the rice genome contains 35 members of the *AS2/LOB* family (Yang *et al.*, 2006). Although the structural conservation of the AS2/LOB domain predicts a ubiquitous role(s) for this domain in proteins of the AS2/LOB family, the molecular and cellular functions of the members of this family have not yet been demonstrated.

Previous studies have suggested that some members of the *AS2/LOB* gene family, namely, *ASL1/LBD36*, *ASL4/LOB* and *ASL5/LBD12*, are involved in the development of various lateral organs from the meristem in Arabidopsis plants (Shuai *et al.*, 2002; Nakazawa *et al.*, 2003; Chalfun-Junior *et al.*, 2005). It has already been reported that *ASL1/LBD36* and *AS2* play partially redundant roles in the determination of cell fate in flower petals (Chalfun-Junior *et al.*, 2005). In maize, loss-of-function mutations, namely, *indeterminate gametophyte 1* (*ig1*) and *ramosa 2* (*ra2*), in genes that encode proteins with an AS2/LOB domain, affect the development of the embyo sac and branching patterns, respectively (Bortiri *et al.*, 2006; Evans, 2007). Recently, mutations in the *ASL19/LBD30* gene of Arabidopsis and a mutation in the *DEGENERATED HULL1* (*DH1*) gene, one of the members of this family found in rice, have also been reported (Borghi *et al.*, 2007; Li *et al.*, 2008).

The *Crown rootless1/Adventitous rootless1* (*Crl1/Arl1*) gene of rice, and the *rootless concerning crown and seminal roots* (*rtcs*) gene of maize encode proteins with AS2/LOB domains that exhibit high degrees of identity, in terms of their respective amino acid sequences, with those of ASL16/LBD29 and ASL18/LBD16 of Arabidopsis. The transcription of each of these genes increases in response to exogenous auxin, and the gene products act downstream of some *AUXIN RESPONSE FACTOR* (*ARF*) genes, and function in the formation of crown roots and lateral roots (Inukai *et al.*, 2005; Liu *et al.*, 2005; Taramino *et al.*, 2007; Okushima *et al.*, 2005, 2007). The expression of *ASL9/LBD3* is induced by cytokinin (Kiba *et al.*, 2005; Naito *et al.*, 2007).

Although sequences of cDNAs for 25 of the 42 genes in this family are available in the DDBJ/GenBank/EBL databases, cDNAs of 17 of the 42 members have not yet been submitted. To clarify the roles of members of the *AS2/LOB* family in Arabidopsis, and, also, the functional relationships among *AS2* and other family members, we cloned and characterized cDNAs that covered at least the putative coding regions of all 42 members of the family, and systematically investigated the patterns of transcription of the corresponding genes in Arabidopsis plants. We did not analyze *LBD34* (Shuai *et al.*, 2002), because the predicted amino acid sequence of its AS2/LOB domain does not include the conserved glycine residue and the leucine-zipper-like sequence that are essential for the functions of AS2 (Iwakawa *et al.*, 2002; YM, unpublished data). The results of our analysis showed that there are eight groups, of two or three genes each, the respective members of which exhibit high degrees of identity in terms of their entire respective amino acid sequences. Moreover, we found that no *ASL/LBD* gene encodes a protein with structural similarity to the C-terminal half of AS2. Patterns of transcription differed among members of the family, but some similarities were apparent. To examine the functional similarities among the AS2/LOB domains of AS2 and other family members, we performed domain-swapping experiments. Our results showed that the AS2/LOB domains of ASL/LBD proteins cannot replace that of AS2 during leaf development, even if only a few amino acid residues differ between the AS2/LOB domain of AS2 and those of the other members of the family. Moreover, our results indicated that *AS2* is a single-copy gene in Arabidopsis.

## Results

### All members of the *AS2/LOB* gene family are expressed in Arabidopsis

Using RT-PCR, with appropriate sets of gene-specific primers, we isolated cDNAs for 17 members of the *AS2/LOB* gene family, for which sequences were not available in the databases. To confirm the gene structures of the 25 family members for which cDNAs had already been submitted to the databases, we also isolated the corresponding cDNAs. All the cDNA sequences were submitted to the DDBJ/GenBank/EMBL databases (Table S1). In the case of the 17 genes for which no cDNA sequences had been submitted to the databases, the amino acid sequences deduced from 16 of the cDNAs cloned in the present study were identical to those predicted from genes in the databases. The results of our analysis of the cDNA for *ASL22/LBD31* showed that the splice acceptor site of this gene was located 15 bp upstream of the site that had been predicted by TIGR analysis of the Arabidopsis genome, to yield five additional amino acid residues in the leucine-zipper-like motif in the AS2/LOB domain.

**Figure 1:**
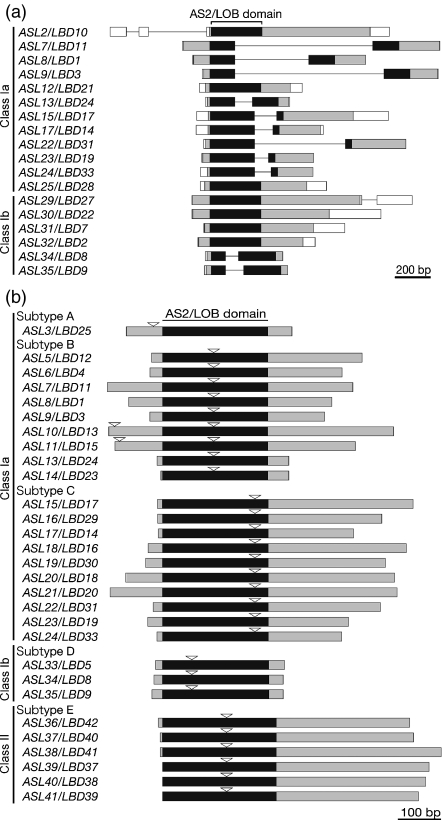
Schematic representations of *ASL/LBD* genes. (a) The exon–intron organization of 17 newly identified *ASL/LBD* genes, and that of *ASL15/LBD17*. Boxes and lines indicate the exons and introns, respectively, that were deduced from genomic and cDNA sequences. Open and filled (gray and black) boxes represent untranslated and translated regions, respectively. The regions corresponding to AS2/LOB domains are indicated by black boxes. (b) The five subtypes of genes in the *AS2/LOB* family, based on positions of introns in coding regions. Filled (gray and black) boxes and open triangles indicate coding regions and positions of introns, respectively. Black boxes show the regions that correspond to AS2/LOB domains. *ASL/LBD* genes with no introns in their coding regions are not shown.

The nucleotide sequences of the cDNAs for the 25 genes that had previously been submitted to the databases were identical to the sequences determined in the present study. However, the cDNA for *ASL15/LBD17* that we cloned extended to a further-upstream region, and included a previously unidentified methionine codon, indicating that the predicted initiation codon of the *ASL15/LBD17* gene is located 171 bp upstream of that of the previously characterized gene. Figure 1a shows the gene organization of the 17 newly characterized members of the family and *ASL15/LBD17*.

Four genes, namely, *ASL25/LBD28*, *ASL30/LBD22*, *ASL31/LBD7* and *ASL32/LBD2*, among the newly identified genes contained no introns, as judged from genomic and cDNA sequences. To investigate the transcription of these genes, we amplified the DNA sequence that corresponded to the 3′ region of the cDNA of each of these genes, and determined the nucleotide sequences of all the amplified products. Our results showed that all the cDNAs that corresponded to these genes contained clusters of deoxyadenylate nucleotides, suggesting that all of these genes are actively transcribed.

### Conservation of the positions of introns in the *AS2/LOB* gene family

Members of the *AS2/LOB* family have been classified into two classes, and at least three subclasses, class Ia, class Ib and class II, on the basis of a phylogenetic tree that was generated from the predicted amino acid sequences of the encoded AS2/LOB domains (Iwakawa *et al.*, 2002). A comparison of the sequences of the cloned cDNAs with those of the corresponding genes revealed that 29 genes contained introns in their coding regions, and these genes could be divided into five subtypes on the basis of the positions of the introns in the coding regions (subtypes A–E; Figure 1b).

With the exception of *ASL3/LBD25*, *ASL10/LBD13* and *ASL11/LBD15*, these *ASL/LBD* genes contained single introns in the regions that encoded their respective AS2/LOB domains. The introns in the genes of each respective subtype were positioned at the same location in the regions that encoded the AS2/LOB domains. Subtype A included only *ASL3/LBD25*, which contained a single intron in the region upstream of the AS2/LOB domain, and this gene belonged to class Ia. Subtypes B and C belonged to class Ia, and subtype D belonged to class Ib. All the members of class II had single introns at the same position, and were classified as subtype E. *ASL10/LBD13* and *ASL11/LBD15* were of subtype B, with additional introns in the region upstream of the AS2/LOB domain. The amino acid sequences of the AS2/LOB domains of predicted ASL/LBD proteins of the same subtype were similar to one another (Iwakawa *et al.*, 2002). This observation suggests that the positions of introns in the regions that encode the AS2/LOB domains have been conserved among the *ASL/LBD* genes that belong to the same respective clades on the phylogenetic tree.

### Comparison of the C-terminal halves of genes in the *AS2/LOB* family

**Figure 2:**
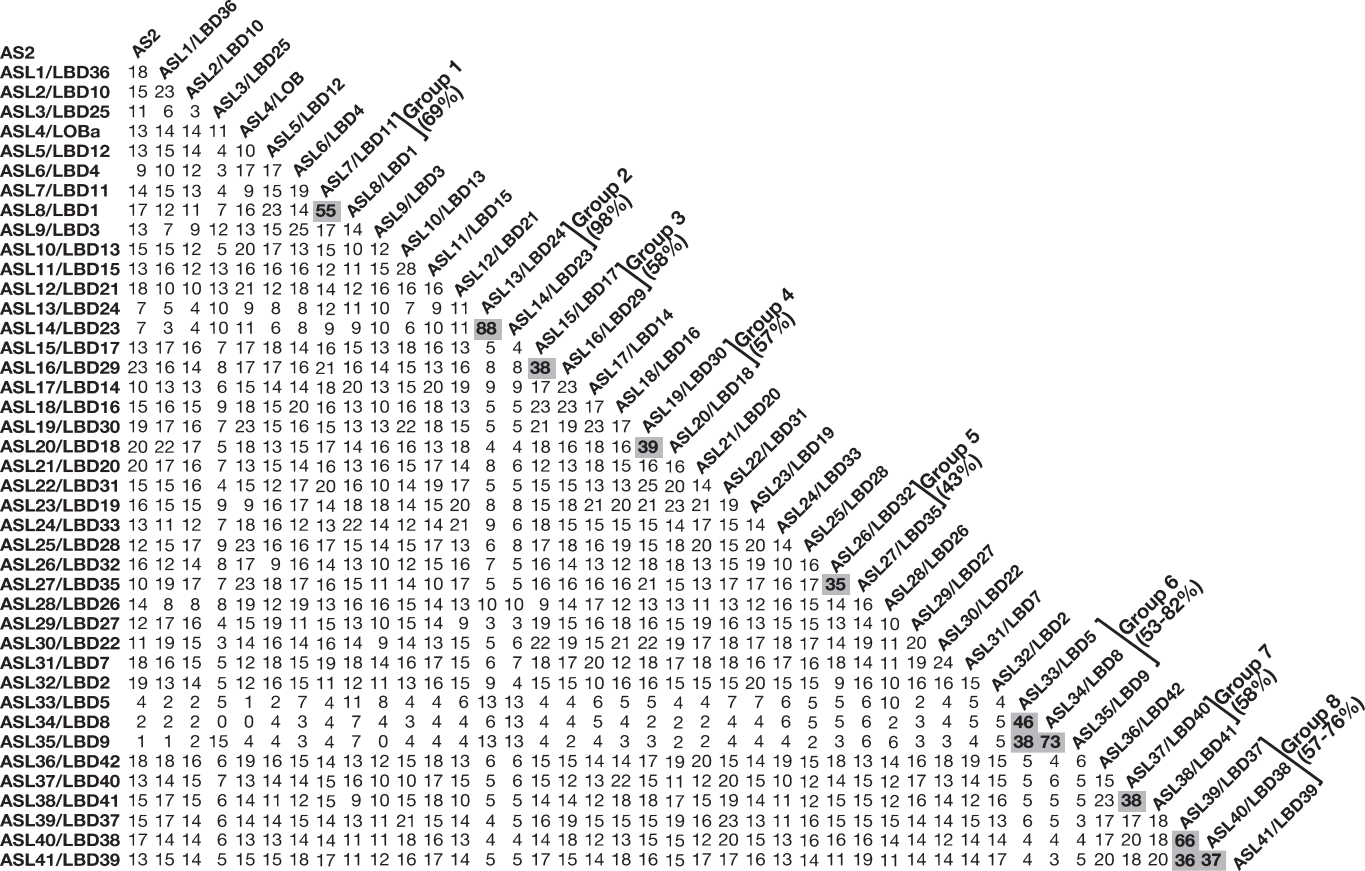
Comparison of the predicted amino acid sequences of the C-terminal halves of products of genes in the *AS2/LOB* family. Each number indicates the identity, as a percentage, between the predicted amino acid sequences of indicated proteins, as determined with the Maximum Matching program of GENETYX-MAC ver. 13. Percentages greater than 35% are shaded. Numbers in percentages under group designations indicate the identities between entire amino acid sequences.

We compared the deduced amino acid sequences of the C-terminal halves (the regions downstream of the respective AS2/LOB domains) of all members of the gene family (Figure 2). No deduced product of any *ASL/LBD* gene exhibited any significant similarity, in terms of amino acid sequence, to the C-terminal half of AS2.

There were eight groups of predicted ASL/LBD proteins, each of which included two or three members, that exhibited more than 35% identity, in terms of amino acid residues, in their C-terminal halves (shaded gray in Figure 2): ASL7/LBD11 and ASL8/LBD1 (group 1; 55%); ASL13/LBD24 and ASL14/LBD23 (group 2; 88%); ASL15/LBD17 and ASL16/LBD29 (group 3; 38%); ASL19/LBD30 and ASL20/LBD18 (group 4; 39%); ASL26/LBD32 and ASL27/LBD35 (group 5; 35%); ASL33/LBD5, ASL34/LBD8 and ASL35/LBD9 (group 6; 38–73%); ASL37/LBD40 and ASL38/LBD41 (group 7; 38%); and ASL39/LBD37, ASL40/LBD38 and ASL41/LBD39 (group 8; 36–66%). These predicted proteins were similar to one another not only in their C-terminal halves, but also in the AS2/LOB domains of their N-terminal halves (Iwakawa *et al.*, 2002). The members of groups 2 and 6, namely, ASL13/LBD24 plus ASL14/LBD23, and ASL33/LBD5 plus ASL34/LBD8 plus ASL35/LBD9, had C-terminal halves that included fewer than 16 amino acid residues.

### Duplication in the genome of genes in the *AS2/LOB* family

**Figure 3:**
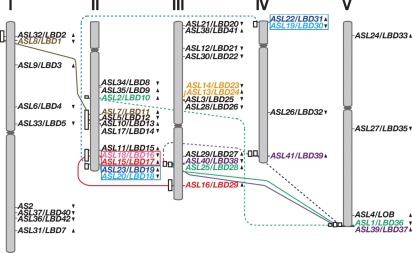
Chromosomal positions and duplication of genes in the *AS2/LOB* family in Arabidopsis. Gray bars and dark-gray ellipses show the chromosomes and the positions of centromeres, respectively. The chromosome number is given at the top of each chromosome. Black arrowheads next to the names of genes indicate the directions of transcription. White bars on the left of chromosomes indicate duplicated segments that contain pairs of putative duplicated genes in the *AS2/LOB* family. Each set of putative duplicated genes is shown in a single color. Solid and dashed lines link each set of duplicated genes, the genomic positions of whidch are encompassed by recently and old duplicated segments, respectively. Members of three pairs of *ASL/LBD* genes that are located next to one another are boxed. Members of groups 1, 2, 3, 4 and 8 that are considered to be sets of duplicated genes are shown in brown, orange, red, light blue and purple, respectively.

We investigated a possible relationship between the genetic divergence of members of the *AS2/LOB* family and duplication events in the Arabidopsis genome. We examined the chromosomal locations and the duplicated segments in which genes of the *AS2/LOB* family are found, using data from the Arabidopsis Genome Initiative (2000) and from the report by Blanc *et al.* (2003) (Figure 3). We found that the genes are distributed over all five chromosomes, with the exception of the short arm of chromosome II. Four sets of genes (*ASL1/LBD36* and *ASL25/LBD28*; *ASL7/LBD11* and *ASL8/LBD1*; *ASL16/LBD29* and *ASL18/LBD16*; and *ASL39/LBD37* and *ASL40/LBD38*) constitute pairs of duplicated genes in blocks of chromosomes that were duplicated approximately 24–40 million years ago. Four other pairs of genes (*ASL1/LBD36* and *ASL2/LBD10*; *ASL19/LBD30* and *ASL20/LBD18*; *ASL39/LBD37* and *ASL41/LBD39*; and *ASL40/LBD38* and *ASL41/LBD39*) constitute pairs of duplicated genes in the older blocks that were duplicated in the more distant past.

Although Blanc *et al.* (2003) did not define three sets of genes (*ASL13/LBD24* and *ASL14/LBD23*; *ASL15/LBD17* and *ASL16/LBD29*; and *ASL22/LBD31* and *ASL23/LBD19*) as pairs of duplicated genes, our analysis revealed that these genes are closely related to one another (see below). *ASL13/LBD24* and *ASL14/LBD23* were located close to each other on chromosome III, and their nucleotide sequences and deduced amino acid sequences were 99 and 98% identical, respectively (Figure 2), suggesting a duplication event. ASL15/LBD17 was the most similar to ASL16/LBD29 among all of the deduced proteins of the AS2/LOB family of Arabidopsis (Figure 2), and *ASL15/LBD17* was located next to *ASL18/LBD16*, which formed a pair with *ASL16/LBD29*. Thus, *ASL15/LBD17* and *ASL16/LBD29* can be considered as a duplicated gene pair. ASL22/LBD31 was most similar to ASL23/LBD19, in terms of the respective AS2/LOB domains (68%), among all of the AS2/LOB proteins of Arabidopsis. *ASL22/LBD31* and *ASL23/LBD19* were located next to *ASL19/LBD30* and *ASL20/LBD18*, respectively. The latter two genes were considered to be a duplicated gene pair. Therefore, *ASL22/LBD31* and *ASL23/LBD19* can also be considered to be a duplicated gene pair. The family members belonging to the five groups described in the previous section appeared, therefore, to represent duplicated genes in each combination.

No traceable history of duplication was evident for 22 genes, even though their genomic positions were encompassed by duplicated blocks. Five genes, namely, *AS2*, *ASL26/LBD32*, *ASL27/LBD35*, *ASL30/LBD22* and *ASL33/LBD5*, were not found in duplicated blocks.

### Patterns of accumulation of transcripts of genes in the *AS2/LOB* family in Arabidopsis

Our examination of transcripts of members of the *AS2/LOB* gene family provided evidence that all members of the family are actively transcribed (Figure 1a and Table S1).

**Figure 4:**
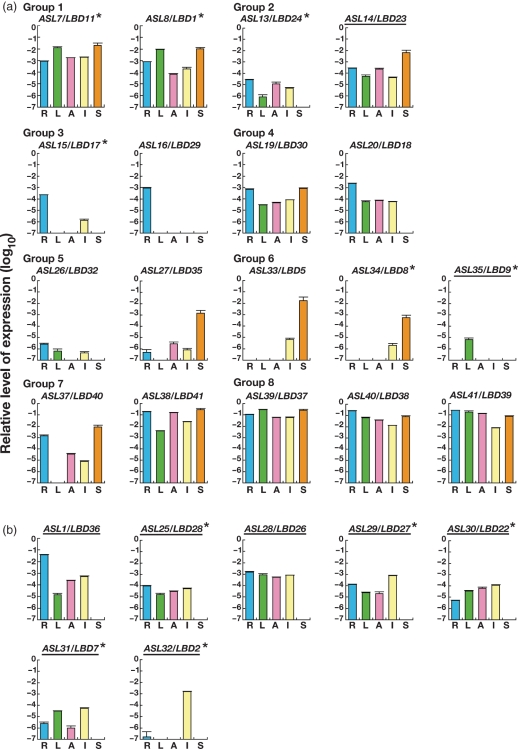
Expression profiles of various members of the *AS2/LOB* family. (a) Profiles of the eight groups with high degrees of sequence identity. (b) Profiles of newly analyzed *AS2/LOB* genes that were not included in the eight groups above. Genes that were newly analyzed in this study are underlined. Asterisks indicate the genes for which cDNAs were determined in this study. The levels of gene expression in the *AS2/LOB* family were determined by quantitative real-time RT-PCR. Poly(A)^+^ RNAs were prepared from mature roots (R), mature leaves (L) and shoot apices (A) of 20-day-old plants, and from inflorescence organs with inflorescence stems, cauline leaves, flower buds and flowers (I), and developing siliques (S), of 35-day-old plants. The relative levels of transcripts of genes in the *AS2/LOB* family were normalized by reference to the level of transcripts of the *EF1-α* gene. Means + SD are shown (*n*= 3).

Members of each of the eight groups might be expected to have common or redundant functions if they are expressed in the same tissue(s) and/or organ(s). To examine the accumulation in various tissues of the transcript of each member of the *AS2/LOB* family, we performed quantitative real-time RT-PCR with poly(A)^+^ RNAs that had been prepared from mature roots, mature leaves, shoot apices, inflorescence organs with inflorescence stems, cauline leaves, flower buds and flowers, and developing siliques. Figure 4 shows the accumulation profiles of the transcripts of the genes belonging to the eight groups (Figure 4a), including transcripts of genes for which no expression data were available in published reports or databases (Figure 4b).

Transcripts of both *ASL7/LBD11* and *ASL8/LBD1* were detected mainly in mature leaves and siliques (Figure 4a; group 1). Transcripts of *ASL15/LBD17* and *ASL16/LBD29* were predominantly present in roots (Figure 4a; group 3). Transcripts of *ASL39/LBD37*, *ASL40/LBD38* and *ASL41/LBD39* were detected in all organs (Figure 4a; group 8).

The genes in other groups seemed to be expressed differently. High levels of the transcripts of *ASL14/LBD23*, *ASL27/LBD35*, *ASL33/LBD5* and *ASL34/LBD8* were detected in siliques (Figure 4a; groups 2, 5 and 6). Transcripts of *ASL19/LBD30* and *ASL20/LBD18* were detected in roots, and only transcripts of *ASL19/LBD30* were found in siliques (Figure 4a; group 4). Transcripts of *ASL35/LBD9* were only found in mature leaves (Figure 4a; group 6). The levels of transcripts of *ASL38/LBD41* were much higher than those of *ASL37/LBD40*, the transcripts of which were undetectable in mature leaves (Figure 4a; group 7).

High levels of transcripts of *ASL1/LBD36* were detected in roots (Figure 4b). Transcripts of *ASL32/LBD2* were present only in inflorescence organs. Transcripts of *ASL25/LBD28*, *ASL28/LBD26*, *ASL29/LBD27*, *ASL30/LBD22* and *ASL31/LBD7* were detected in all organs, with the exception of siliques.

### The *AS2/LOB* domains of members of the *AS2/LOB* family cannot replace the functions of the *AS2/LOB* domain of AS2 in leaf development

To investigate whether the AS2/LOB domains of ASL/LBD proteins are functionally analogous with the AS2/LOB domain of AS2, we replaced the AS2/LOB domain of AS2 with those of other members of the family, and tested the chimeric proteins for their ability to reverse the abnormal phenotype of the *as2-1* mutant. We chose *ASL1/LBD36*, *ASL2/LBD10*, *ASL3/LBD25* and *ASL4/LOB* as representative members of the gene family that are most closely related to *AS2*, and we chose *ASL15/LBD17*, *ASL18/LBD16*, *ASL23/LBD19* and *ASL37/LBD40* as distantly related members.

**Figure 5:**
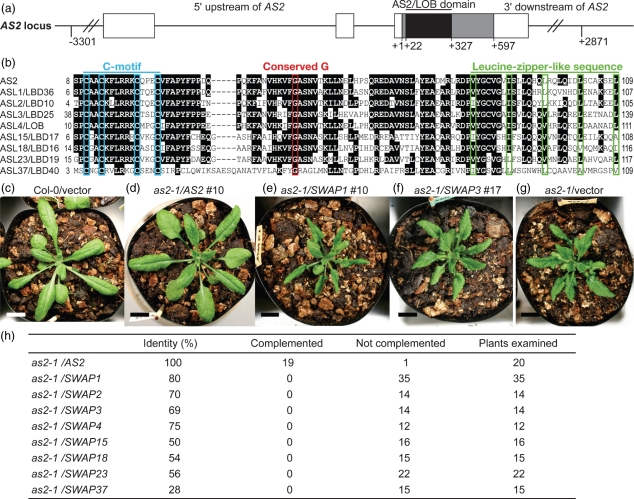
Replacement of the AS2/LOB domain of AS2 with that of various ASL/LBD proteins. (a) Schematic representation of the genomic fragment that includes the *AS2* locus. Open and filled (gray and black) boxes represent untranslated and translated regions of the *AS2* gene, respectively. The region corresponding to the AS2/LOB domain is indicated by a black box. Numbers below the line and boxes indicate the nucleotide positions of the translated region (from +1 to +597 nt), the region corresponding to the AS2/LOB domain (from +22 to +327 nt) and, positions upstream (−3301 nt) and downstream (+2871 nt) of translated regions of the *AS2* gene, which were used for the domain-swapping experiments. (b) Comparison of the amino acid sequences of AS2/LOB domains. The sequence from residue 8 to residue 109 of AS2 is aligned with sequences from corresponding regions of the indicated ASL/LBD proteins. Amino acid residues conserved in more than five proteins are indicated by white characters on a black background. The consensus sequence of the C-motif, the conserved glycine residue and the hydrophobic residues in the leucine-zipper-like sequence are indicated by blue, red and green boxes, respectively. (c–g) Gross morphology of various *as2-1/SWAP* transgenic plants. The name and the line number of each transgenic plant are indicated above the respective panel. (c) A 38-day-old wild-type plant into which the empty vector had been introduced; (d) 30-day-old and (e) and (f) 33-day-old transgenic plants; (g) a 41-day-old *as2-1* plant into which the empty vector had been introduced. (h) Classification of the *as2-1/SWAP* transgenic plants. The extent of the identity between the deduced amino acid sequence of the AS2/LOB domain of the indicated protein, and that of AS2, is shown as a percentage. Scale bars: 10 mm.

The genomic DNA region [nucleotide positions (nt) from +22 to +327] encoding the AS2/LOB domain in the *AS2* genomic segment (from −3301 to +2871 nt) was replaced by individual AS2/LOB domain-encoding DNA fragments of the *ASL/LBD* genes mentioned above (Figure 5a,b), to generate chimeric genes, which were designated as *SWAP1*, *SWAP2*, *SWAP3*, *SWAP4*, *SWAP15*, *SWAP18*, *SWAP23* and *SWAP37*, respectively. These chimeric constructs, as well as the genomic *AS2* segment, were introduced into *as2-1* mutant plants. We obtained 20 lines of *as2-1*/*AS2* transgenic plants (Figure 5d,h). Among these transgenic lines, 19 lines produced flat and symmetric leaves, as did wild-type plants. Only one line still produced downwardly curling leaves.

We obtained 35, 14, 14, 12, 16, 15, 22 and 15 lines of *as2-1/SWAP1*, *as2-1/SWAP2*, *as2-1/SWAP3*, *as2-1/SWAP4*, *as2-1/SWAP15*, *as2-1/SWAP18*, *as2-1/SWAP23* and *as2-1/SWAP37* transgenic plants, respectively. None of these *SWAP* lines showed any evidence of complementation (Figure 5e,f,h), with the exception that the leaves, which were reduced in length in the proximal–distal direction in *as2-1* plants, were slightly closer to the wild-type length in 17 out of the 35 *as2-1*/SWAP1 transgenic plants (Figure 5e). These results suggest that the AS2/LOB domains of ASL/LBD proteins other than AS2 cannot functionally replace the AS2/LOB domain of AS2 in leaf development.

**Figure 6:**
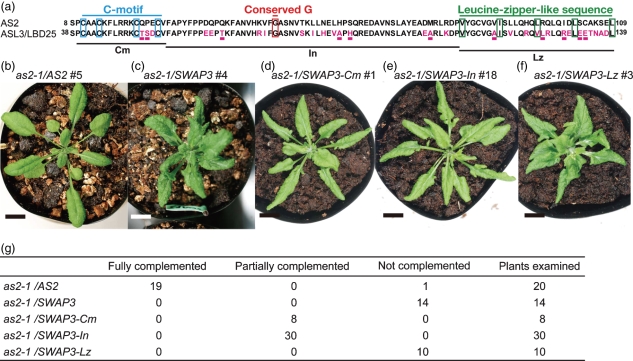
Replacement of three regions in the AS2/LOB domain of AS2 with the corresponding regions of ASL3/LBD25. (a) Comparison of the amino acid sequences of the AS2/LOB domains of AS2 and ASL3/LBD25. The sequence from residue 8 to residue 109 of AS2 is aligned with the sequence from the corresponding region of ASL3/LBD25. The consensus sequences of the C-motif, the conserved glycine residue, and the hydrophobic residues in the leucine-zipper-like sequence are indicated by blue, red and green boxes, respectively. Amino acid residues (either similar or dissimilar) that are not identical with those in AS2 are indicated by pink characters, and the dissimilar amino acid residues are underlined. Black lines below the sequences show the C-motif (Cm), internal (In) and the leucine-zipper-like sequence (Lz) regions, respectively. (b–f) Gross morphology of various *as2-1/SWAP* transgenic plants. The name and the line number of each transgenic plant are indicated above the respective panel. (b) and (c) 33-day-old transgenic plants; (d), (e) and (f) 31-day-old transgenic plants. (g) Classification of the *as2-1/SWAP* transgenic plants with respect to complementation of the *as2-1* mutation. The results of experiments with *as2-1*/*gAS2* and *as2-1/SWAP3* were obtained in Figure 4 (h) Scale bars: 10 mm.

To investigate which amino acid residues in the AS2/LOB domain of AS2 are crucial for the functions of AS2, we performed additional domain-swapping experiments. We chose *ASL3/LBD25*, one of the members of class Ia, the members of which are most closely related to *AS2*. The DNA sequence encoding the AS2/LOB domain of AS2 was split into three regions (Figure 6a): the Cm region that encodes the C-motif (from +22 to +72 nt); the Lz region that encodes the leucine-zipper-like sequence (from +241 to +327 nt); and the In region that encodes the internal regions, including the conserved glycine residue between Cm and Lz (from +73 to +240 nt). Each DNA segment encoding a region of the AS2/LOB domain of AS2 was replaced by the corresponding DNA fragment that encoded the AS2/LOB domain of ASL3/LBD25 to generate chimeric genes, which were designated as *SWAP3-Cm*, *SWAP3-In* and *SWAP3-Lz* (Figure 6a). The Cm, In and Lz regions from both AS2 and ASL3/LBD25 contained 17, 56 and 29 amino acid residues, respectively. According to calculations made by the ClustalW multiple sequence alignment program (Larkin *et al.*, 2007; http://www.ebi.ac.uk/tools/clustalw2), these regions include two, four and five dissimilar residues, respectively (underlined in Figure 6a).

We introduced these three chimeric constructs into *as2-1* mutant plants, and obtained 8, 30 and 10 lines of *as2-1/SWAP3-Cm*, *as2-1/SWAP3-In* and *as2-1/SWAP3-Lz* transgenic plants, respectively (Figure 6d–g). None of the constructs fully reversed the phenotype caused by the *as2* mutation, in contrast to the genomic *AS2* segment, which did restore a normal phenotype (Figure 6b). The length of *as2* leaves in the proximal–distal direction was restored to wild-type values by the *SWAP3-Cm* and *SWAP3-In* constructs, but the downward curling and the rough surface of leaves were unaffected (Figure 6d,e). The rates of asymmetric formation of deep leaf lobes and leaflet-like structures were also scarcely affected in *as2-1/SWAP3-Cm* and *as2-1/SWAP3-In* transgenic plants, as compared with *as2-1* plants. None of the *as2-1/SWAP3-Lz* transgenic plants showed evidence of complementation of the *as2-1* mutation, and the same was true of *as2-1/SWAP3* transgenic plants (Figure 6f,c). These results indicate that the dissimilar amino acid residues in the AS2/LOB domain of AS2 are important for the functions of AS2, and that the five dissimilar residues in the Lz region of AS2 are the most critical.

We used RT-PCR to determine the levels of transcripts of the transgenes in the various lines of transgenic plants, and found that the respective levels of each transcript were similar to one another (data not shown).

## Discussion

In the present study, we found that transcripts of all of the members of the AS2/LOB family accumulate at different respective levels in Arabidopsis plants, and have a variety of transcriptional profiles in the whole plant (Figures 4 and S1). We also found eight groups, with two or three members each, of ASL/LBD proteins with high degrees of identity among their entire amino acid sequences (Figure 2). No member of the family exhibited significant similarity, at the amino acid level, to the C-terminal half of AS2 itself. Moreover, domain swapping between AS2 and other members of the family showed that the functions of the AS2/LOB domain in AS2 could not be replaced by those of the AS2/LOB domains of other ASL/LBD proteins (Figure 5). Further analysis with the segmented AS2/LOB domain of ASL3/LBD25 also demonstrated that a few amino acid residues (between two and five) in this domain of AS2 were critical for the functions of AS2 (Figure 6).

### The gene family includes eight groups of members that exhibit strong similarity in terms of their entire respective amino acid sequences

We found eight groups, with two or three members each, of ASL/LBD proteins with high degrees of identity to one another in terms of their entire amino acid sequences (Figure 2). The members of five of the eight groups were considered to be duplicated genes in each combination, suggesting evolutionary conservation (Figure 3).

In some groups, the members had similar transcriptional profiles (Figure 4; *ASL7/LBD11* and *ASL8/LBD1*; *ASL15/LBD17* and *ASL16/LBD29*; and *ASL39/LBD37*, *ASL40/LBD38* and *ASL41/LBD39*). The genes in the other groups, seemed to be expressed differently from one another (Figure 4; *ASL13/LBD24* and *ASL14/LBD23*; *ASL19/LBD30* and *ASL20/*LBD18; *ASL26/LBD32* and *ASL27/LBD35*; *ASL33/LBD5*, *ASL34/LBD8* and *ASL35/LBD9*; and *ASL37/LBD40* and *ASL38/LBD41*). The members of each group might have overlapping functions in those organs in which they are expressed together.

We obtained T-DNA insertion lines of 16 *ASL/LBD* genes, including members of four groups, from the Arabidopsis Biological Resource Center (ABRC, http://www.biosci.ohio-state.edu/~plantbio/Facilities/abrc/abrchome.htm) and the Nottingham Arabidopsis Stock Centre (NASC, http://arabidopsis.info), and we then investigated the gross morphology of the aerial parts of these insertion lines (Figure S2). There were no apparent differences between these insertion lines and wild-type plants, suggesting functional redundancy within the AS2/LOB family or insignificant function in the aerial parts of plants.

### Dissimilar amino acid residues in the AS2/LOB domains are important for characteristic functions of members of the AS2/LOB family

The results of domain-swapping experiments with the AS2/LOB domains from various ASL/LBD proteins demonstrated that the AS2/LOB domain of AS2 cannot be replaced by those of other members of the gene family to achieve appropriate functioning of AS2 (Figure 5). Although AS2 and ASL1/LBD36 have strongly conserved amino acid sequences in their AS2/LOB domains (80% identity), the AS2/LOB domain of ASL1/LBD36 was unable to act, within AS2, to reverse the mutant phenotype of the leaf lamina in *as2* plants. The shortness of *as2* leaves in the proximal–distal direction was, however, overcome to a slight extent by this construct (Figure 5e). This result seems to be consistent with the previous report that AS2 and ASL1/LBD36 act in a partially redundant manner to control the determination of cell fate in Arabidopsis petals (Chalfun-Junior *et al.*, 2005). The AS2/LOB domains encoded by other *ASL/LBD* genes (inserted into AS2 without its own AS2/LOB domain) did not complement the *as2* mutation to any detectable extent. These observations suggest functional diversity among the members of the *AS2/LOB* family, even when there is strong structural similarity among the AS2/LOB domains.

Domain-swapping experiments using three regions of the AS2/LOB domain of ASL3/LBD25 showed that no region of ASL3/LBD25 was fully able to reverse the abnormal *as2* phenotype. Transgenic plants with the Lz region of ASL3/LBD25 did not show any evidence of complementation: neither the length of leaves in the proximal–distal direction nor the asymmetric shapes of leaves were restored (Figure 6f,g). The Lz regions of AS2 and ASL3/LBD25 contain 14 different and 15 identical residues. Nine out of the 14 different residues are similar, and five residues are dissimilar, to one another (underlined in Figure 6a). The latter five residues appear to be critical for the functions of AS2. Note that these dissimilar residues are located at positions next to or next but one to hydrophobic residues (L, I or V) that are characteristic of the leucine zipper. These five residues might be involved in protein–protein interactions that are responsible for the functional specificity of AS2.

Transgenic plants with the Cm region of ASL3/LBD25 only partially resembled wild-type plants: the length of leaves in the proximal–distal direction was restored to that of a wild-type plant, although the asymmetric shape and the rough surface of the leaves were not restored (Figure 6d). The respective Cm regions of ASL3/LBD25 and AS2 contain three different and 14 identical residues. One out of the three different residues is similar, and two residues are dissimilar (underlined in Figure 6a). The two dissimilar residues (Q21 and P22) appear to be important for the functions of AS2 in the formation of a symmetric and flat leaf lamina.

Transgenic plants with the In region of ASL3/LBD25 also only partially resembled wild-type plants: the phenotype of the transgenic plants was similar to that of the plants with the Cm region of ASL3/LBD25 (Figure 6d,e). The respective In regions of ASL3/LBD25 and AS2 contain 14 different and 42 identical residues. Ten out of the 14 different residues are similar, and four residues are dissimilar (underlined in Figure 6a). These four residues might be important for functions of AS2 in the formation of a symmetric and flat leaf lamina.

The above results of the domain-swapping experiments suggest that the leaf growth in the proximal–distal direction and the formation of a symmetric and flat leaf lamina are independently regulated by the AS2/LOB domain of AS2.

Note that the locations of the above-defined dissimilar amino acid residues are significantly conserved among the AS2/LOB domains of most members of the family (Figure 5b; Iwakawa *et al.*, 2002). In particular, the Cm regions contain clusters of three of the so-called dissimilar residues at identical respective locations: between the third and fourth conserved cysteine residues (see Figure 5b). These observations suggest that, although the amino acid sequences of AS2/LOB domains are strongly conserved in all family members, the dissimilar amino acid residues might be responsible for the characteristic functions of each member of the family.

## Experimental procedures

### Plant strains and growth conditions

*Arabidopsis thaliana* ecotype Col-0 (CS1092) and the *as2-1* (CS3117) mutant were obtained from ABRC. We outcrossed *as2-1* with Col-0 three times, and used the progeny for our experiments. The T-DNA insertion lines of 16 *ASL/LBD* genes were obtained from the ABRC or NASC (Table S2). For the analysis of plants, seeds were sown on soil or on gellan gum-solidified MS medium. After two days at 4°C in darkness, plants were transferred to a regimen of white light at 50 μmol m^−2^ sec^−1^ for 16 h, followed by darkness for 8 h, daily, at 22°C, as described previously (Semiarti *et al.*, 2001). The ages of the plants are given as number of days after vernalization.

### Isolation of RNA and synthesis of cDNA

We used 10-day-old seedlings and 35-day-old plants for cloning cDNA, and we used roots, leaves and shoot apices from 20-day-old plants, and inflorescence organs with inflorescence stems, cauline leaves, flower buds and flowers, and siliques from 35-day-old plants for quantitative real-time RT-PCR. All plants and organs were frozen immediately in liquid nitrogen after harvest, and stored at −80°C prior to use. Total RNA was extracted with an RNeasy kit (Qiagen, http://www.qiagen.com), with subsequent treatment with DNase to remove any contaminating genomic DNA. Poly(A)^+^ RNA was isolated with Dynabeads (Dynal Biotech, http://www.invitrogen.com/dynal). Reverse transcription was performed with a First-Strand cDNA Synthesis Kit (GE Healthcare, http://www.gehealthcare.com) and oligo dT primers with or without the additional adaptor sequence 5′-CTGATCTAGAGGTACCGGATCC-3′.

### Cloning of cDNA

We amplified cDNAs by PCR using primer sets specific for each gene. Primers were designed on the basis of regions outside of the predicted coding sequences, for the amplification of cDNAs of 17 genes for which no cDNA sequences had been submitted to the databases, and on the basis of untranslated regions, for the amplification of cDNAs of 25 genes for which sequences of cDNA clones had been submitted to the databases. Primers for cloning into the pDONR221 vector (Invitrogen, http://www.invitrogen.com) contained the additional adaptor sequence 5′-AAAAGCGGCT-3′ for forward primers and 5′-AGAAAGCTGGGT-3′ for reverse primers, and second PCRs were performed with products of the first PCR as template, the attB1 adaptor primer 5′-GGGGACAAGTTTGTACAAAAAAGCAGGCT-3′ and the attB2 adaptor primer 5′-GGGGACCACTTTGTACAAGAAAGCTGGGT-3′. Products of PCR were gel-purified, cloned into the pDONR221 vector or the pGEM-T vector (Promega, http://www.promega.com), and then sequenced. The sequences of primers that are not given here can be found in Table S3.

### Database search and analysis of nucleotide and amino acid sequences

For information about the cDNAs and expressed sequence tags (ESTs) that corresponded to genes of the *AS2/LOB* family, we searched the TAIR database using the Arabidopsis Genome Initiative (AGI) ID. The positions of exons and introns in individual genes of the *AS2/LOB* family were determined by comparison of cDNA sequences with the corresponding genomic DNA sequences. Numbers that indicate matching percentages (identity) among predicted amino acid sequences were determined with the Maximum Matching program in genetyx-mac v13 (GENETYX, http://www.sdc.co.jp/genetyx). The amino acid sequences of the AS2/LOB domain of AS2, and that of ASL3/LBD25, were compared by the ClustalW multiple sequence alignment program (Larkin *et al.*, 2007; http://www.ebi.ac.uk/tools/clustalw2).

### Chromosomal locations and analysis of duplication of genes in the *AS2/LOB* family

The chromosomal location of each member of the *AS2/LOB* family was determined with the Chromosome Map Tool at TAIR (http://www.arabidopsis.org/jsp/ChromosomeMap/tool.jsp). The location of each gene in relation to major chromosomal duplication events in the Arabidopsis genome was determined with tools provided at http://wolfe.gen.tcd.ie/athal/dup and defined by Blanc *et al.* (2003).

### Quantitative real-time RT-PCR

Primer sets corresponding to genes in the *AS2/LOB* family and the *EF1-α* (*ELONGATION FACTOR1-α*; At1g07940) gene were designed with Primer Express 2.0 software (Applied Biosystems, http://www.appliedbiosystems.com). Sequences were confirmed with the BLAST program to ensure that primers would allow amplification of unique and appropriate cDNA segments (see Table S4). PCR was performed in the presence of the dye SYBR Green (Applied Biosystems), which is specific for double-stranded DNA, according to the manufacturer’s instructions. Amplification was monitored in real time with a Real-Time PCR System (model 7500; Applied Biosystems). To quantify the levels of cDNAs, and, thus, of transcripts, standards were prepared from serial dilutions of *AS2/LOB* and *EF1-α* cDNAs. The standards were analyzed in parallel with cDNAs prepared from various plant organs, and were used to generate standard curves. Upon completion of amplification reactions, melting curves were generated to verify that a single product had been amplified in each case. The quantification of each sample of cDNA was performed in triplicate. The results were normalized by reference to results for *EF1-α*.

### Construction of plasmids

To construct pGAS2ΔAS2d, we inserted the oligonucleotide 5′-CATGCAGGTGCCCGTCGACGGGCACCTG-3′ into the *Nco*I site of pGPTAS2-linker4, which was constructed by the insertion of the 5′ and coding region of *AS2* (from −3301 to +21 nt), an *Nco*I site, and the coding and 3′ region of *AS2* (from +328 to +2871 nt), into the *Apa*I/*Eco*RV sites of pGreen0029 (Hellens *et al.*, 2000). cDNA fragments corresponding to the AS2/LOB domains of AS2, ASL1/LBD36, ASL2/LBD10, ASL3/LBD25, ASL4/LOB, ASL5/LBD12, ASL15/LBD17, ASL18/LBD16, ASL23/LBD19 and ASL37/LBD40 were amplified using specific primers, digested by *Aar*I, and inserted into the *Aar*I site of pGAS2ΔAS2d to generate pG*gAS2*, pG*SWAP1*, pG*SWAP2*, pG*SWAP3*, pG*SWAP4*, pG*SWAP15*, pG*SWAP18*, pG*SWAP23* and pG*SWAP37*, respectively (see Table S5). cDNA fragments corresponding to the Cm region (from +22 to +72 nt), the In region (from +73 to +240 nt) and the Lz region (from +241 to +327 nt) of the AS2/LOB domain of AS2 were replaced with cDNA fragments that encoded the corresponding regions of ASL3/LBD25. To generate pG*SWAP3-Cm*, pG*SWAP3-In* and pG*SWAP3-Lz*, cDNA fragments of *AS2* and *ASL3/LBD25* were amplified with specific primers, digested by *Aar*I, and then inserted into the *Aar*I site of pGAS2ΔAS2d (see Table S5).

### Transformation

We introduced plasmids pG*AS2*, pG*SWAP1*, pG*SWAP2*, pG*SWAP3*, pG*SWAP4*, pG*SWAP15*, pG*SWAP18*, pG*SWAP23*, pG*SWAP37*, pG*SWAP3-Cm*, pG*SWAP3-In* and pG*SWAP3-Lz* into *Agrobacterium tumefaciens* strain GV3101. Whole plants (*as2-1*) were then transformed by vacuum infiltration, as described by Bechtold and Pelletier (1998). Transgenic plants were selected on MS medium that contains 35 μg ml^−1^ kanamycin.

## SUPPORTING INFORMATION

**Figure S1.** Profiles of the expression of genes of the *AS2/LOB* family in various organs.

**Figure S2.** Phenotypes of mature plants (38–43-days old), with the insertion mutations indicated.

**Table S1.** Summary of cDNAs that corresponded to members of the *AS2/LOB* gene family in Arabidopsis.

**Table S2.** T-DNA insertion lines corresponding to the 16 indicated *ASL/LBD* genes examined in this study.

**Table S3.** Primers used for cDNA cloning and sequence analysis.

**Table S4.** Primers used for quantitative RT-PCR.

**Table S5.** Primers used for the preparation of *SWAP* constructs.
