# Supplementary figures and images for: Characterization of genes in the ASYMMETRIC LEAVES2/LATERAL ORGAN BOUNDARIES (AS2/LOB) family in Arabidopsis thaliana, and functional and molecular comparisons between AS2 and other family members
Source: Plant J. 2009 Mar 2;58(3):525–37. doi: 10.1111/j.1365-313X.2009.03797.x (PMC2721968; doi:10.1111/j.1365-313X.2009.03797.x)

Relative level of expression ( $\log_{10}$ )

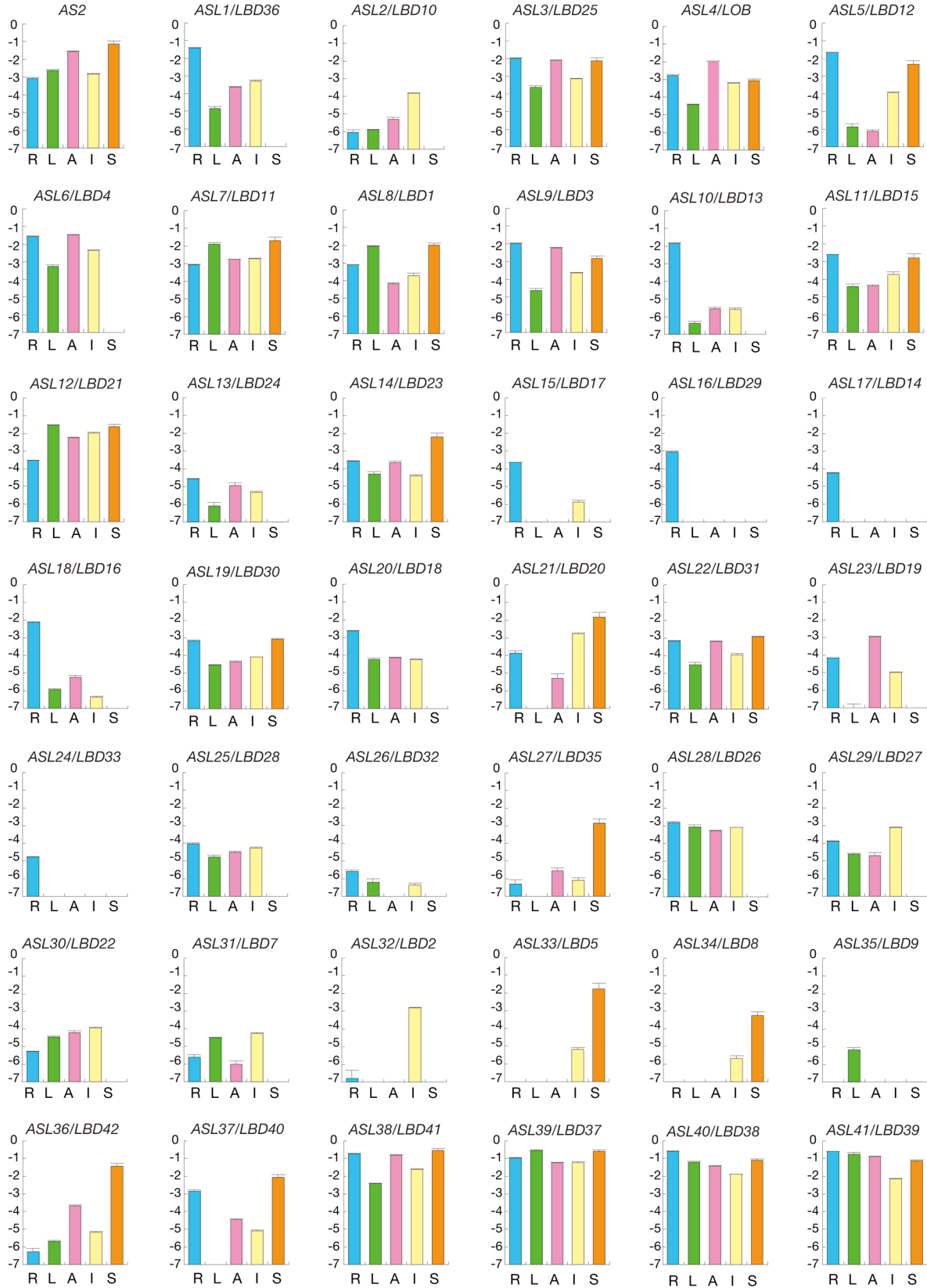

Supplement: Supplementary file 1 [file tpj0058-0525-SD1.pdf]

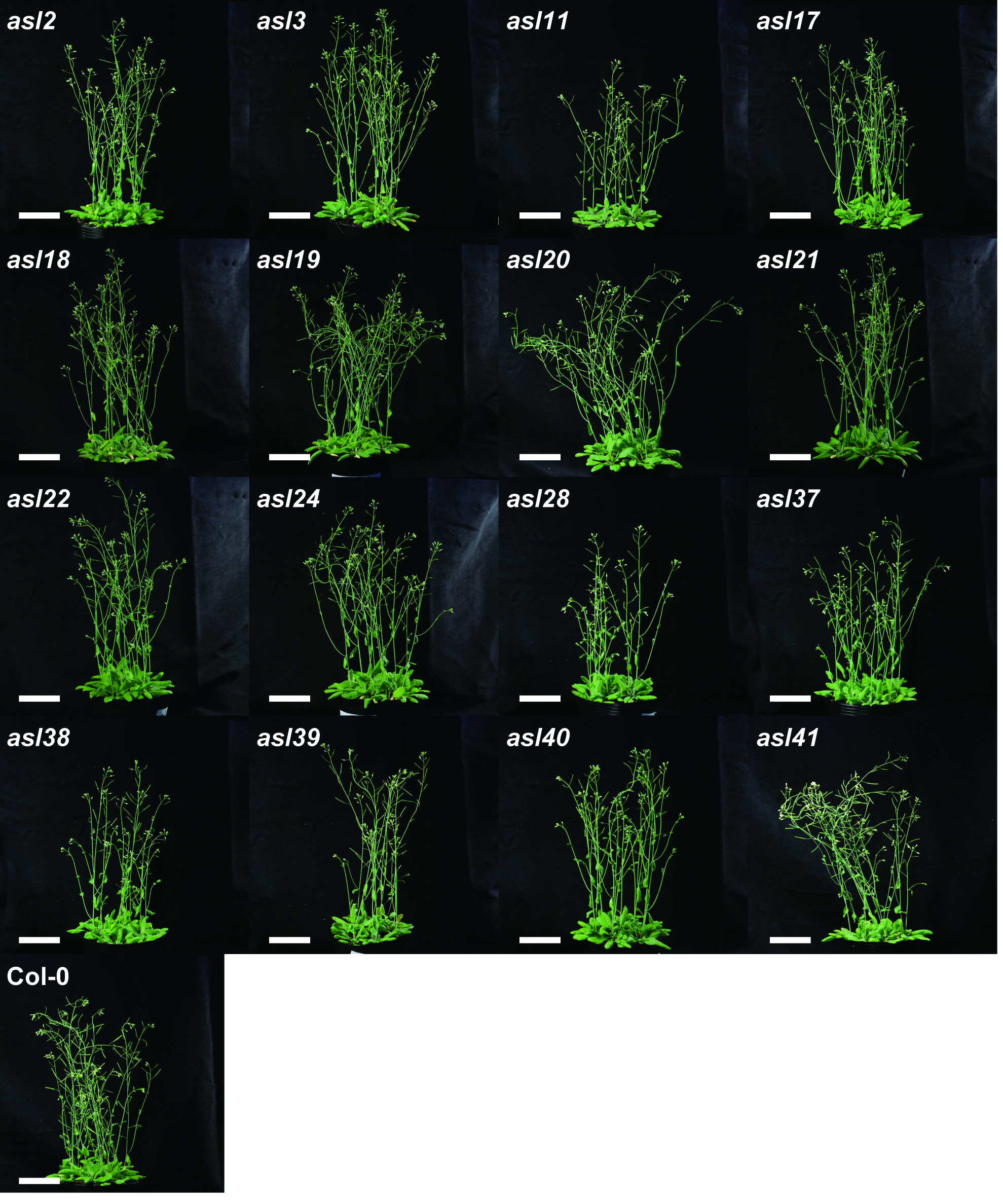

Supplement: Supplementary file 2 [file tpj0058-0525-SD2.tif]
