# Supplementary material for: Characterization of genes in the ASYMMETRIC LEAVES2/LATERAL ORGAN BOUNDARIES (AS2/LOB) family in Arabidopsis thaliana, and functional and molecular comparisons between AS2 and other family members
Source: Plant J. 2009 Mar 2;58(3):525–37. doi: 10.1111/j.1365-313X.2009.03797.x (PMC2721968; doi:10.1111/j.1365-313X.2009.03797.x)
Supplement: Supplementary file 3 [file tpj0058-0525-SD3.doc]

**Table S1.** Summary of cDNAs that corresponded to members of the *AS2/LOB* gene family in Arabidopsis.

|  | Gene name | Arabidopsis Genome Initiative ID | Accession no. of cloned cDNA | Accession no. of previously submitted cDNA sequences ^a, b^ | No. of ESTs ^c^ |
| --- | --- | --- | --- | --- | --- |
| Class Ia | |  |  |  |  |
|  | *AS2* | At1g65620 | to be submitted | AB080802 (AS2), AF447887 (LBD6), AY299251, BT025163 | 20 |
|  | *ASL1/LBD36* | At5g66870 |  | AB164303 (ASL1), BX832456 | 3 |
| * | *ASL2/LBD10* | At2g23660 |  |  | 2 |
|  | *ASL3/LBD25* | At3g27650 |  | AB164304 (ASL3), AF447892 ( LBD25), AY088878 | 9 |
|  | *ASL4/LOB* | At5g63090 |  | AB164305( ASL4), AF447897 ( LOBa), AF447898 ( LOBb), AF447899 ( LOBc), AF447900 ( LOBd), BT025745 | 2 |
|  | *ASL5/LBD12* | At2g30130 |  | AB164306 ( ASL5 ), AY086960 | 2 |
|  | *ASL6/LBD4* | At1g31320 |  | BT015067, BT015665, BX818457 |  |
| * | *ASL7/LBD11* | At2g28500 |  |  | 2 |
| * | *ASL8/LBD1* | At1g07900 |  |  | 1 |
| * | *ASL9/LBD3* | At1g16530 |  |  | 4 |
|  | *ASL10/LBD13* | At2g30340 |  | AF345338, AF447888 (LBD13), AY429384, BT026052 | 33 |
|  | *ASL11/LBD15* | At2g40470 |  | AF447889 (LBD15), DQ653051, DQ446613 (LBD15), AY088856, BT025288 | 5 |
| * | *ASL12/LBD21* | At3g11090 |  |  | 18 |
| * | *ASL13/LBD24* | At3g26660 |  |  |  |
|  | *ASL14/LBD23* | At3g26620 |  | DQ056609 |  |
|  | *ASL15/LBD17* | At2g42440 |  | AY074651 |  |
|  | *ASL16/LBD29* | At3g58190 |  | AF447893 (LBD29), BT025282 | 2 |
| * | *ASL17/LBD14* | At2g31310 |  |  |  |
|  | *ASL18/LBD16* | At2g42430 |  | AF410334, AF447890 (LBD16),  AF345339, BT000866 | 44 |
|  | *ASL19/LBD30* | At4g00220 |  | AF432232 (LBD30), BT026407,  DQ446787 (LBD30), DQ653168 | 4 |
|  | *ASL20/LBD18* | At2g45420 |  | AF447891 (LBD18), BT025716, BX820287 | 4 |
|  | *ASL21/LBD20* | At3g03760 |  | AK118291, BT008589 | 7 |
| * | *ASL22/LBD31* | At4g00210 |  |  |  |
| * | *ASL23/LBD19* | At2g45410 |  |  | 2 |
| * | *ASL24/LBD33* | At5g06080 |  |  |  |
| * | *ASL25/LBD28* | At3g50510 |  |  |  |
|  | *ASL26/LBD32* | At4g22700 |  | DQ056654 | 1 |
|  | *ASL27/LBD35* | At5g35900 |  | DQ056696 |  |
|  | *ASL28/LBD26* | At3g27940 |  | AK118484, BT003673 | 5 |
| Class Ib | |  |  |  |  |
| * | *ASL29/LBD27* | At3g47870 |  |  | 2 |
| * | *ASL30/LBD22* | At3g13850 |  |  |  |
| * | *ASL31/LBD7* | At1g72980 |  |  |  |
| * | *ASL32/LBD2* | At1g06280 |  |  |  |
|  | *ASL33/LBD5* | At1g36000 |  | DQ056480 |  |
| * | *ASL34/LBD8* | At2g19510 |  |  |  |
| * | *ASL35/LBD9* | At2g19820 |  |  |  |
| Class II | |  |  |  |  |
|  | *ASL36/LBD42* | At1g68510 |  | AF447896 ( LBD42), BX817744, BX817752, BX841914 | 1 |
|  | *ASL37/LBD40* | At1g67100 |  | BT010943, BT011650, BX816029 | 14 |
|  | *ASL38/LBD41* | At3g02550 |  | AF447895 ( LBD41), AY086061, AY090370, AY122896, BX822366 | 71 |
|  | *ASL39/LBD37* | At5g67420 |  | AF447894 ( LBD37), AY087998, BT024477, BX830145, BX830821, BX831254, BX831283, BX841687 | 73 |
|  | *ASL40/LBD38* | At3g49940 |  | AY085761, BT002449, BT006287, BX824099 | 43 |
|  | *ASL41/LBD39* | At4g37540 |  | AY072144, AY087291, AY122976 | 38 |

^a^Accession numbers. of cDNA sequences previously submitted to GenBank. ^b^Sequences of underlined clones were submitted to GenBank as full-length cDNAs. ^c^ Number of EST clones found for each gene. *cDNAs were determined in the present study and submitted to GenBank.

**Table S2.** T-DNA insertion lines corresponding to the 16 indicated *ASL/LBD* genes and examined in this study.

| Gene name | AGI No. | seed |
| --- | --- | --- |
| *ASL2/LBD10* | At2g23660 | SALK_073853 |
| *ASL3/LBD25* | At3g27650 | SALK_033840 |
| *ASL11/LBD15* | At2g40470 | SALK_019954 |
| *ASL17/LBD14* | At2g31310 | SALK_093993 |
| *ASL18/LBD16* | At2g42430 | SALK_095791 |
| *ASL19/LBD30* | At4g00220 | SALK_024953 |
| *ASL20/LBD18* | At2g45420 | SALK_038125 |
| *ASL21/LBD20* | At3g03760 | SALK_054710C |
| *ASL22/LBD31* | At4g00210 | SALK_021150 |
| *ASL24/LBD33* | At5g06080 | SAIL_95_H10 |
| *ASL28/LBD26* | At3g27940 | SALK_065331 |
| *ASL37/LBD40* | At1g67100 | SALK_140608 |
| *ASL38/LBD41* | At3g02550 | SALK_144556 |
| *ASL39/LBD37* | At5g67420 | SALK_057939 |
| *ASL40/LBD38* | At3g49940 | SALK_037369 |
| *ASL41/LBD39* | At4g37540 | SALK_049910C |

**Table S3.** Primers used for cDNA cloning and sequence analysis.

| Gene name |  | Primer (5'-3') |
| --- | --- | --- |
| *AS2* | Fw | GTTGTTCTTTCTCTCCGTTTCC |
| *AS2* | Rv | GGGCGGCCGCTCAAGACGGATCAACAGTACGGC |
| *ASL1/LBD36* | Fw | CTGGGTTTTCTCAGAAACCCTAGAC |
| *ASL1/LBD36* | Rv | TTTGTTTCGGGATGGAGTGGA |
| *ASL2/LBD10* | Fw | AAAAAGCAGGCTTTTTTGATAGTGTTGTGTCTTAAGTGG |
| *ASL2/LBD10* | Rv | AGAAAGCTGGGTTTTTGTTCTCCTAAATGCATTTAGGTC |
| *ASL2/LBD10* | Rv | TCTCCTCTCTCCAACATCAT |
| *ASL3/LBD25* | Fw | AAAAAGCAGGCTGCCTTTTGCCCCTCTCTCTCTATATC |
| *ASL3/LBD25* | Rv | AGAAAGCTGGGTAAATATTCCTTGACCTCTCCCATG |
| *ASL4/LOB* | Fw | AAAAAGCAGGCTTTGTCTTTTGCTCTTTCTCCTTCC |
| *ASL4/LOB* | Rv | AGAAAGCTGGGTATAATATCCCACACACAGTCCATG |
| *ASL4/LOB* | Fw | TCAAAGAACCTAAAGATCTA |
| *ASL5/LBD12* | Fw | AAAAAGCAGGCTTCTGCTCTCAAAGCTCAAGAGAAC |
| *ASL5/LBD12* | Rv | AGAAAGCTGGGTGAACATTAATTATTATTATTATTATTAC |
| *ASL5/LBD12* | Fw | GTTGCAGGAGCTACCAGTTC |
| *ASL6/LBD4* | Fw | AAAAAGCAGGCTCACAGCTTTCTCGATAACTGACAATC |
| *ASL6/LBD4* | Rv | AGAAAGCTGGGTTAACAAAGATCCCTCCTCGGTTGG |
| *ASL6/LBD4* | Rv | CTATCCGGGCATAGTCCGTG |
| *ASL7/LBD11* | Fw | AAAAAGCAGGCTCCCCCAAAATATTTTGAGAATCATGC |
| *ASL7/LBD11* | Rv | AGAAAGCTGGGTGAGCTAATAATACCATAAGTTATG |
| *ASL8/LBD1* | Fw | AAAAAGCAGGCTCCCATAATTCACAGTTTTTGCGTTG |
| *ASL8/LBD1* | Rv | AGAAAGCTGGGTAGCGAAGTAGTCAAAATCTTAGTAATG |
| *ASL8/LBD1* | Rv | TGAGCTTTTGCAAGTTGTGC |
| *ASL9/LBD3* | Fw | AAAAAGCAGGCTTTGCTCTCAATTCTCAAAAACTCTC |
| *ASL9/LBD3* | Rv | AGAAAGCTGGGTTGCAAAGATATGATAGAGTGCTAAC |
| *ASL10/LBD13* | Fw | AAAAAGCAGGCTGATACTCTAAAATAAACTTATAAATAGC |
| *ASL10/LBD13* | Rv | AGAAAGCTGGGTAGCTTATAATAAGGGCTATAATAG |
| *ASL10/LBD13* | Fw | CTTTGGTGCAAGCAACGTCT |
| *ASL11/LBD15* | Fw | AAAAAGCAGGCTCCTCTTTAAGAGTCACTTGACTTT |
| *ASL11/LBD15* | Rv | AGAAAGCTGGGTTTAGTCACCGGATCACAAATTATC |
| *ASL11/LBD15* | Fw | GATCAAAAGAGAAGCAGATG |
| *ASL12/LBD21* | Fw | AAAAAGCAGGCTAAAACGAGAGCCTAACATTTACCAC |
| *ASL12/LBD21* | Rv | AGAAAGCTGGGTAAGAGATATATGATAGAGAGATAAGGC |
| *ASL12/LBD21* | Fw | GTCTCTGCAGAAGAAGATG |
| *ASL12/LBD21* | Rv | TCCAAAGGGGACACTTGGCT |
| *ASL13/LBD24* | Fw | AAAAAGCAGGCTGTGTTGTTTGTTTATATTTGTTTCTGAG |
| *ASL13/LBD24* | Rv | AGAAAGCTGGGTCAGAGGAGATTTTATTGATGAAACTG |
| *ASL14/LBD23* | Fw | AAAAAGCAGGCTTTTATATTTGTGTCTGAGAAAATCTGAG |
| *ASL14/LBD23* | Rv | AGAAAGCTGGGTGAGGAGAATTTATTGTTGAAACTGAG |
| *ASL15/LBD17* | Fw | GGGGACAAGTTTGTACAAAAAAGCAGGCTCCTAAGCTAATGCTCCTCCA |
| *ASL15/LBD17* | Rv | GGGGACCACTTTGTACAAGAAAGCTGGGTTTCGACGACTTGACTCCGAT |
| *ASL16/LBD29* | Fw | AAAAAGCAGGCTATTCGACCTTTAATATCAAGAACCG |
| *ASL16/LBD29* | Rv | AGAAAGCTGGGTTCTAAAAAAAACATCTTGAGGATG |
| *ASL16/LBD29* | Rv | AACCCATATTCTTGATAGAA |
| *ASL17/LBD14* | Fw | AAAAAGCAGGCTCGTCACTAGAAGAGTATAAGTAATG |
| *ASL17/LBD14* | Rv | AGAAAGCTGGGTGAAAGATTGTAAGGTTCCATCTCTG |
| *ASL18/LBD16* | Fw | AAAAAGCAGGCTCATTTCTTGTTCTTTTTCCACCATCAC |
| *ASL18/LBD16* | Rv | AGAAAGCTGGGTATTGATGATAGCATGTGAACATGG |
| *ASL18/LBD16* | Rv | CGGATTGTTAGGGTTTACAT |
| *ASL19/LBD30* | Fw | AAAAAGCAGGCTGCATCATAATCTCGATGAGTTAAATCC |
| *ASL19/LBD30* | Rv | AGAAAGCTGGGTAACAATTTAAATACGAATTCGACC |
| *ASL19/LBD30* | Rv | ACGCGCCAGTGCTTGGAGCT |
| *ASL20/LBD18* | Fw | AAAAAGCAGGCTAAAAAGCAGGCTGAGTGGGGAGATAGAGTAACAACTC |
| *ASL20/LBD18* | Rv | AGAAAGCTGGGTAGAAAGCTGGGTCTAATAACAATGCGGAGATGTATCG |
| *ASL20/LBD18* | Rv | CGAGCCCCACGGCGACAG |
| *ASL21/LBD20* | Fw | AAAAAGCAGGCTAGATATCGTTTATCTCGCAAGAAAC |
| *ASL21/LBD20* | Rv | AGAAAGCTGGGTCATTCAAATATTTCACGACAACTC |
| *ASL21/LBD20* | Rv | TGGCATTGGCTGCAACGAAT |
| *ASL22/LBD31* | Fw | AAAAAGCAGGCTCCAAACAAAACGTTACGTCTTTGAC |
| *ASL22/LBD31* | Rv | AGAAAGCTGGGTATAAATGTCGCAAAGGTTAACATGG |
| *ASL22/LBD31* | Rv | TATTGTCTTTGCTATCGACA |
| *ASL23/LBD19* | Fw | AAAAAGCAGGCTCTCTCTTGCTCTCTCTCTTTACTTG |
| *ASL23/LBD19* | Rv | AGAAAGCTGGGTTACATAAATTCTTGACTTGTAGTTTGAC |
| *ASL24/LBD33* | Fw | AAAAAGCAGGCTAACCAACGTGATTTGTCTCAATCTAAC |
| *ASL24/LBD33* | Rv | AGAAAGCTGGGTTAGTTCGCGTCGAATTTTGTTGTGC |
| *ASL24/LBD33* | Rv | TTCAAAACACTCTCGTCGAC |
| *ASL25/LBD28* | Fw | AAAAAGCAGGCTCTAGAAATGATCAAAAATGTACAATTAC |
| *ASL25/LBD28* | Rv | AGAAAGCTGGGTAAAGCTTACCATTGTTATCAAGATTC |
| *ASL26/LBD32* | Fw | AAAAAGCAGGCTGGGTTAAGAGAGAAAATAAAACAAAG |
| *ASL26/LBD32* | Rv | AGAAAGCTGGGTTTGATTTTCAGAAAATGGAAGTATCTC |
| *ASL27/LBD35* | Fw | AAAAAGCAGGCTGTTTTTTGGTTACTAGGTAAGCGAG |
| *ASL27/LBD35* | Rv | AGAAAGCTGGGTACCAAATTATTATGGGAATTGAGATGC |
| *ASL28/LBD26* | Fw | AAAAAGCAGGCTTGGAGAAAAACACAATTGAGAATCG |
| *ASL28/LBD26* | Rv | AGAAAGCTGGGTTTAAAAGCATATTTGGTTCCAAAG |
| *ASL29/LBD27* | Fw | AAAAAGCAGGCTAGAGTCTCTTGTTCTGTTCCCATCG |
| *ASL29/LBD27* | Rv | AGAAAGCTGGGTAAGGAAAAAACCCCTACCTTGAGGC |
| *ASL29/LBD27* | Rv | GTTGTGTTTCGGAAACTGGT |
| *ASL30/LBD22* | Fw | AAAAAGCAGGCTGAACTCGCCAAACCCGTCAAACAAG |
| *ASL30/LBD22* | Rv | AGAAAGCTGGGTAGCTACGGTTCAAGAAAATGTAAGCTG |
| *ASL31/LBD7* | Fw | AAAAAGCAGGCTCTTCCATATATCCCAAAACATAAACC |
| *ASL31/LBD7* | Rv | AGAAAGCTGGGTAGACTAAAAACCTGAAAGGAACTAC |
| *ASL32/LBD2* | Fw | AAAAAGCAGGCTTCAAGTTTCCGTTACTAGGGACATG |
| *ASL32/LBD2* | Rv | AGAAAGCTGGGTTTTTTTTTCCGTCAAACATTATAGTTAC |
| *ASL33/LBD5* | Fw | AAAAAGCAGGCTCTTCAATTACTTAATCAAAATTTATCTC |
| *ASL33/LBD5* | Rv | AGAAAGCTGGGTGATGCATAAATAATATATGCTACAATG |
| *ASL34/LBD8* | Fw | AAAAAGCAGGCTCTCAATCAATTTTTATTTCTAAAAGAAC |
| *ASL34/LBD8* | Rv | AGAAAGCTGGGTGATGCATAAATAATATATGCTACAATG |
| *ASL35/LBD9* | Fw | AAAAAGCAGGCTTCTTTCAATTAACAAATTTATAGTTGTATTTACAC |
| *ASL35/LBD9* | Rv | AGAAAGCTGGGTACAACACAATATATCTCAACACACTATATCATTAG |
| *ASL36/LBD42* | Fw | AAAAAGCAGGCTTCCTTATAAATTCAAATTGTTCAC |
| *ASL36/LBD42* | Rv | AGAAAGCTGGGTGTTTTTTTTTCTCCTTTTATATAG |
| *ASL36/LBD42* | Fw | TTGTGGTCGGATCGTGAACC |
| *ASL37/LBD40* | Fw | AAAAAGCAGGCTAACACATTTTGTTTTTTGTTTCCCATC |
| *ASL37/LBD40* | Rv | AGAAAGCTGGGTATTTATGAAGAACACCGAACATGC |
| *ASL38/LBD41* | Fw | AAAAAGCAGGCTGCGGTTCTGGGAAAATAAAGGAAG |
| *ASL38/LBD41* | Rv | AGAAAGCTGGGTCAGCCAATTCTTGAATTAATCTCTG |
| *ASL38/LBD41* | Fw | ACACAATCCTTTAAAGCTAT |
| *ASL38/LBD41* | Fw | GCAGCTTTGTCAAGACGCCG |
| *ASL39/LBD37* | Fw | AAAAAGCAGGCTTCCCAGACATCAGCAAGATTCAG |
| *ASL39/LBD37* | Rv | AGAAAGCTGGGTTCGCCGGTCCAATATTTATCCAG |
| *ASL39/LBD37* | Fw | TGCTGGTCTCATGTCCTTT |
| *ASL40/LBD38* | Fw | AAAAAGCAGGCTACTCTCTCTCTTTCTCTTTCTTGTAC |
| *ASL40/LBD38* | Rv | AGAAAGCTGGGTCTCTATTACTTTGGATAATTTCTC |
| *ASL40/LBD38* | Fw | TTGGAATGTTTGTCAAGCGG |
| *ASL41/LBD39* | Fw | AAAAAGCAGGCTGAAACTTGTTTTCTCTTTCCCTTC |
| *ASL41/LBD39* | Rv | AGAAAGCTGGGTAAATATCCATTCCCACTCAAAACC |
| *ASL41/LBD39* | Fw | ATTCTTTGGTCGTGCTGGT |
| *EF 1-alpha* | Fw | TGAGCACGCTCTTCTTGCTTTCA |
| *EF 1-alpha* | Rv | GGTGGTGGCATCCATCTTGTTACA |

Fw and Rv represent forward and reverse primers, respectively.

| **Table S4.** Primers used for quantitative RT-PCR. | |  |
| --- | --- | --- |
| Gene name | Primer 1 (5'-3') | Primer 2 (5'-3') |
| *AS2* | TGCGCCGCTTGCAAAT | AAATAGGGCGCGAATACACATT |
| *ASL1/LBD36* | GGTTCAGTTACGGTTACTGGGTTT | TCGTCGAAGGACTATCAACAAAGT |
| *ASL2/LBD10* | CGGTTACAGCCACGGGTTT | GATGGTCCAGACAACGAACCA |
| *ASL3/LBD25* | TTGTCTCGGTGGTGAAACGA | CCGCTAATTTTCTCAAAGAGTGATG |
| *ASL4/LOB* | GGCAAGTCCGGTGATGAATC | GATCATGACCATTGTTCCAAGGA |
| *ASL5/LBD12* | ACATCAATAACTGCAACACCAACA | TGCTTGGTGAGGCAAAGTTAGA |
| *ASL6/LBD4* | CCAGGGCACGGACTATGC | TGTGGACTCACTTGCTTCGAA |
| *ASL7/LBD11* | CATTTGAAAGCGGCGATGA | CCTCAAGGAACCCCAAATCA |
| *ASL8/LBD1* | ACATGGACCAACAACAGAAACAA | TTGATGAGTTGGTGTTGGACTTG |
| *ASL9/LBD3* | CGTTACTTTCCCCTCGAACAAA | CCGGCATCATCCGTATACG |
| *ASL10/LBD13* | ACGACCACCAAGGTTGTTGTC | ACGGAGATGGCAAAGAAACG |
| *ASL11/LBD15* | ACTCCCAAGTTGCCGGATTT | AGGATGAGCCGTTGTAGGTTGT |
| *ASL12/LBD21* | CACTCGACTACTTGCCCATTCTG | GCAGCAAGCTCAGGAGAATCA |
| *ASL13/LBD24* | CCAAACTTAGCCAAACCCAAAA | CAGAGGAGATTTTATTGATGAAACTG |
| *ASL14/LBD23* | CCAAACTTAGCCAAACCCATAT | GAGGAGAATTTATTGTTGAAACTGAG |
| *ASL15/LBD17* | CATCATGACGTCGTGCTACCA | TCCGCCAGCTCCTGTGA |
| *ASL16/LBD29* | GGAAATGTGCAAAGGGATGTG | GTGAATGGCTGCAAAGTGTGA |
| *ASL17/LBD14* | TGGACTCAGACTTCGACCCAAT | TTCATTTGAGCTACAAGGAGACTCA |
| *ASL18/LBD16* | GCCACAGAGCTCGCTAGAAGA | TCGCACGTTGGCTGTTGT |
| *ASL19/LBD30* | CCACGACCGTCTGATCATCTC | ACCGCCTCCGCCCATAT |
| *ASL20/LBD18* | TCGATCAAACGACGTCTTCTTC | TTGGTGACGTGGATCAATGAA |
| *ASL21/LBD20* | AGAACATTCGTTGCAGCCAAT | TCTTCCTCATCCTCATGCTGATAA |
| *ASL22/LBD31* | TCTTTGGCCTGGAAAATCCA | GCCTCCGGTCAAATACTTAGTGA |
| *ASL23/LBD19* | CCTCTACAGTCGCCACAACAAA | CGGTTCCATCGCATATTCATT |
| *ASL24/LBD33* | TCTCGCCTTACTTCAGCTACGA | TGCGCCGAAGACTTTGTG |
| *ASL25/LBD28* | CGAGGCTCAACGTTTTGCAT | ACTCTCCTCATCGTGCTGCAT |
| *ASL26/LBD32* | AATGCACCAATGATAATGAATAGCA | TGATGCACCATCAGCTCTATGC |
| *ASL27/LBD35* | TCTGCAAAGAATGAGCTTGCA | TCAGCTGGCATAGGAATACTTGAA |
| *ASL28/LBD26* | CGCATAGATACTACCCGCACACT | GGACACCTCTTCAGCTTTTTGG |
| *ASL29/LBD27* | TGGCTATAGTCCTCCTGCTTATAATACA | TTCTGATCACCCCAAACATTGTT |
| *ASL30/LBD22* | CGCCGCCTGCAAATACC | GGGAAATAAGGAGCAAGAAGACAA |
| *ASL31/LBD7* | CAAATCGATATGTGTCGCTCTCTAG | AGGAATTGCAGCGATATGGAA |
| *ASL32/LBD2* | GATTGTCAACGGTGGATATGCA | TGCCTTTGATCGTGCTTTAGATT |
| *ASL33/LBD5* | CACAAAGCCTACCTCCGTGAA | ATCACTAATATAAAGACGAGGCTGGTT |
| *ASL34/LBD8* | AAACAAATGCTTGCAACTTCCA | CACCATACCAAATCCACCACTTAC |
| *ASL35/LBD9* | GAGGTTCGCGTCCAAAGACA | TTCCAAGCGTCACCTTCCA |
| *ASL36/LBD42* | GGCCACTTCACGTTGGAGAA | CCCAAGATTTGGAGAACTTTGATT |
| *ASL37/LBD40* | GACGACGGAGGAACCAAAGA | CACGTGATTTCCACGTGCTAA |
| *ASL38/LBD41* | CGATTTGAAACTTGCGAAAACTC | CCTCCGATTCCGCCTGTAT |
| *ASL39/LBD37* | AAGCAAGCAACACCTTCTTCTACA | CGTCCCGGAGTCCTCTGA |
| *ASL40/LBD38* | TTCTTAACGGCGGTGGATTC | TCCGTACAGATCTCCGAAGCTT |
| *ASL41/LBD39* | TGGCATCAAGACGTTTCAAGAA | TCATCTCCGTCGTGGATCTG |
| *EF 1-alpha* | TGAGCACGCTCTTCTTGCTTTCA | GGTGGTGGCATCCATCTTGTTACA |

**Table S5.** Primers used for the preparation of *SWAP* constructs.

| Primer name | Sequence |
| --- | --- |
| AS2-AS2D-Fw | TCACCATGCGCCGCTT |
| AS2-AS2D-Rv | GAGCTCAGATTTAGCACAGC |
| ASL1/LBD36-AS2D-Fw | TCTCCTTGCGCAGCTTG |
| ASL1/LBD36-AS2D-Rv | GAGCTCTTTCTTAGCGTTTTC |
| ASL2/LBD10-AS2D-Fw | ACACCTTGTGCAGCATGTAAG |
| ASL2/LBD10-AS2D-Rv | AAGCTCTTCTTTAGCAGTGAGAA |
| ASL3/LBD25-AS2D-Fw | TCACCGTGTGCAGCATG |
| ASL3/LBD25-AS2D-Rv | GAGATCAGCATTTGTCTCCTC |
| ASL4/LOB-AS2D-Fv | TCACCATGCGCGGCGT |
| ASL4/LOB-AS2D-Rv | CAAGTCAGCATTAGCTGCG |
| ASL15/LBD17-AS2D-Fw | TCTCCTTGTGGAGCTTGTAAG |
| ASL15/LBD17-AS2D-Rv | TGCTTGTTGCTTTAGAATCTCTAG |
| ASL18/LBD16-AS2D-Fw | TCACCATGCGGTGCATG |
| ASL18/LBD16-AS2D-Rv | TATCTGTGCCTTCATTTGCATG |
| ASL23/LBD19-AS2D-Fw | GGACCATGCGGGGCTT |
| ASL23/LBD19-AS2D-Rv | AAGACGAGCTTGAACATGAGC |
| ASL37/LBD40-AS2D-Fw | ATGAGTTGTAACGGATGTCG |
| ASL37/LBD40-AS2D-Rv | CACGGGTGAGCCTCTC |
| AS2-Cm-Fw | ACGTCAGACTGCGTATTCGCGCCCTATTT |
| AS2-In-Fw | CGACTTAAAGATCCAGTCTACGGCTGCGTC |
| AS2-In-Rv | AAAATAGGGTGCGAATACACATTCCGGTTG |
| AS2-Lz-Rv | TCCAACGCAGCCATACACAGGGTCACGGAGGCGCAT |
| ASL3/LBD25-Cm-Rv | AAATAGGGCGCGAATACGCAGTCTGACGT |
| ASL3/LBD25-In-Fw | CAACCGGAATGTGTATTCGCACCCTATTTT |
| ASL3/LBD25-In-Rv | GACGCAGCCGTAGACTGGATCTTTAAGTCG |
| ASL3/LBD25-Lz-Fw | ATGCGCCTCCGTGACCCTGTGTATGGCTGCGTTGGA |
